# The Immune System and the Antiviral Responses in Chinese Giant Salamander, *Andrias davidianus*

**DOI:** 10.3389/fimmu.2021.718627

**Published:** 2021-10-05

**Authors:** Nan Jiang, Yuding Fan, Yong Zhou, Yan Meng, Wenzhi Liu, Yiqun Li, Mingyang Xue, Jacques Robert, Lingbing Zeng

**Affiliations:** ^1^ Division of Fish Disease, Yangtze River Fisheries Research Institute, Chinese Academy of Fishery Sciences, Wuhan, China; ^2^ Department of Microbiology and Immunology, University of Rochester Medical Center, New York, NY, United States

**Keywords:** Chinese giant salamander, amphibian, antiviral response, immune system, Ranavirus infection

## Abstract

The Chinese giant salamander, belonging to an ancient amphibian lineage, is the largest amphibian existing in the world, and is also an important animal for artificial cultivation in China. However, some aspects of the innate and adaptive immune system of the Chinese giant salamander are still unknown. The Chinese giant salamander iridovirus (GSIV), a member of the Ranavirus genus (family *Iridoviridae*), is a prominent pathogen causing high mortality and severe economic losses in Chinese giant salamander aquaculture. As a serious threat to amphibians worldwide, the etiology of ranaviruses has been mainly studied in model organisms, such as the *Ambystoma tigrinum* and *Xenopus*. Nevertheless, the immunity to ranavirus in Chinese giant salamander is distinct from other amphibians and less known. We review the unique immune system and antiviral responses of the Chinese giant salamander, in order to establish effective management of virus disease in Chinese giant salamander artificial cultivation.

## Introduction

The Chinese giant salamander (*Andrias davidianus*) is the largest and the most primitive urodele amphibian alive worldwide. The Japanese salamander, the American hellbender, and the Chinese giant salamander are few living members belonging to the most ancient amphibian lineage, the family *Cryptobranchidae*, which is distantly related to the *Salamandridae* that emerged some 350 million years ago (1). The morphology of *A*. *davidianus* is thought to have varied very little over this long period of time and, as such, is considered as a living fossil. Populations of *A. davidianus* have declined remarkably within the past 50 years due to habitat loss, environmental change and pollution, overharvesting, infection diseases, and exploitation for food. As a result, *A. davidianus* has been included in the national class II protected species in China and in the list of Appendix I of the Convention on International Trade in Endangered Species of Wild Fauna and Flora (CITES, 2008) (2–5). In parallel to this rapid decline of wild populations, farming of *A. davidianus* has developed rapidly since 2005. In China, approximately 2 million *A. davidianus* are artificially reproduced per year (3).

Due to the development of intensive farming as well as intense international and national trading, emerging infection diseases of *A. davidianus* rapidly increased in prevalence in recent years. The major culprit is a ranavirus pathogen, the Giant Salamander Iridovirus (GSIV), which is also known as *A. davidianus* ranavirus (ADRV). Outbreaks of GSIV disease are threatening both wild and captive bred *A. davidianus*, and mortality rate often reaches 100% in densely populated farms (3, 4, 6–10). GSIV disease first outbroke in 2010 in the Shanxi Province and has rapidly spread to all Chinese giant salamander farming areas, including Hubei, Jiangxi, Hunan, Henan, Sichuan, and Shaanxi provinces (3, 4, 6–8).

As a member of the genus *Ranavirus* (RVs) (family *Iridoviridae*), GSIV is a large icosahedral double-stranded DNA virus. Ranaviruses have emerged as major amphibian pathogens, infecting and causing outbreaks of a wide variety of captive and wild species worldwide (11). The detailed homologous gene comparisons of GSIV with other ranaviruses revealed that GSIV contained 85%, 93%, 94%, 97%, and 99% homologous genes in *Ambystoma tigrinum* virus (ATV), tiger frog virus (TFV), common midwife toad ranavirus (CMTV), frog virus 3 (FV3), and *Rana grylio* virus (RGV) genomes, respectively (8). Moreover, based on the phylogenetic analysis of the concatenated sequences and the Bayesian tree, GSIV is more closely related to CMTV compared to ATV, TFV, FV3, and RGV (12).

With regard to the aquaculture of the Chinese giant salamander and the disease caused by GSIV, besides advances in fundamental comparative immunology, a better understanding of the immune system ontogeny and antiviral immune response is imperative. In contrast to mammals, amphibians have an external development free of maternal influence. As such, their immune system has to develop rapidly within a few days to provide protection to early larval stages against pathogens. In addition, the immune system of anuran amphibians is usually regarded as more efficient than that of urodels. This view is probably due in large part to the disproportionate studies on very few species, the anura *Xenopus laevis* and the urodele *Ambystoma mexicanum* (13, 14). Anurans such as *X. laevis* undergo a remarkable complete metamorphosis that also profoundly remodel their immune system, whereas urodels exhibit a range of developmental transition from partial and incomplete to cryptic metamorphosis (15, 16). Indeed, some salamanders transition through only partial morphological changes such as internalization of the gills, while unlike anurans, they retain their tails in adult stages. In contrast, axolotls exhibit a characteristic called “neoteny” and reach sexual maturity without undergoing metamorphosis (14). *A. davidianus* also undergoes a limited developmental transition during metamorphosis, including the internalization of the gills and the retention of the tail at adult stage.

With regard to the immune system, salamanders such as tiger salamanders and Mexican axolotls are also distinctive compared to frogs, perhaps in part due to their high regeneration capacity (13). Recent advances of genomics and transcriptomics have revealed that many potential orthologues of mammalian innate and adaptive immune response-related genes are present and expressed in urodel species (17, 18). In particular, molecular and functional studies have recently been undertaken in the Chinese giant salamander, which stir misconception of immune capacity of urodel species.

This review presents recent progresses in our knowledge about the organization of immune system and lymphopoiesis of Chinese giant salamander as well as the antiviral responses, which is essential for the protection of wild and artificially cultured Chinese giant salamander.

## Organization of the Chinese Giant Salamander Immune System

The immune systems of jawed vertebrates are categorized into two interconnected arms: innate and adaptive. Unsurprisingly, the two types of immunity are present in the Chinese giant salamander, as well as genes and pathways that define innate and adaptive immunity. The summary of identified immune genes of *A. davidianus* and *X. laevis* is shown in **Table 1**.

**Table 1:** Identified immune genes of *A. davidianus* and *X. laevis*.

		*A. davidianus*	*X. laevis*	Ref.
**Innate immunity**	TLRs	TLR (1–3, 5–8, 10, 13, 19, 20)	TLR (1–9, 12–14, 19, 20)	(17, 21, 22)
	NLRs	NAIP, IPAF, APAF1, NOD proteins (NOD1, NOD2, NOD3, NOD5, CIITA)	Nod 1 and 2, Nod3, NLRX1, CIITA, IPAF, NAIP	(17)
	RLRs	RIG-I, MDA5, LGP2	RIG-I/DDX58	(17, 23)
	AMPs	Cathelicidin-like precursor	Magainin, xenopsin, caerulein, peptide glycine-leucine-amide	(19, 24, 25)
	Complement	C1-9, MASP, CR1, CR2	C1-9, MASP, Bf	(17, 22, 26)
	Signaling adaptor	MyD88,	MyD88, NFKB, IKBB, DAP10, DAP12, TCRgamma	(4, 17, 19)
	Cytokines	IL-1β, TNFα, IL-6, IL-18, I-IFN, IFNγ, IFNβ, IRF3	IL-1β, LTα, LTβ, TNFα, IL6, IFNα	(4, 17, 19, 27, 28)
**Adaptive immunity**	Antigen presentation	MHC I, MHC II, β2M	MHC I, MHC II, DM, β2M, Psm8-10, Tap1, Tap2, Cathepsin L, Tapasin, calreticulin, calnexin	(29–34)
	Antigen receptors	IgH (D, M, Y), IgL (lambda, kappa, type III), TCR β, Rag 1, 2	IgH (M, X, D, F, Y), IgH (λ, κ, σ), TCR α, β, γ, δ, Rag1, Rag 2, AID, TdT	(35–48)
	Accessory molecules	CD8a, CD8b	CD2, CD4, CD8 α, β, BTLA, CD274, CD276, JAM, VTCN1, CD28, CTLA4, HVEM, CD40LG, CD40, MCAM, ICAM, IL-2, 3, 4, 5, 7, IFNγ	(19, 26, 49, 50)
	Signaling molecules	CD3E	LCK, CD3ϵ, CD3ζ, Fyn, Igα	(51, 52)

### Innate Immune System

Innate immunity provides an effective initial defense and is critical to activate adaptive immune responses in amphibians as in mammals (53, 54). Typical innate molecules and pathways include pattern recognition receptors (PRRs), complement system, antimicrobial peptides (AMPs), interferons (IFNs), lectins and inflammatory cytokines, signaling factors, and chemokines (19, 20). Notably, PRR-mediated pathogen detection and recognition is central to the activation of innate and adaptive immune system (20). The molecular interaction of PRRs with pathogen-associated molecular patterns (PAMPs) stimulates immune effector cells, such as dendritic cells, macrophages, and natural killer (NK) cells, and induces related pathways and cytokines, such as Toll-like receptor pathway and the downstream ILs and IFNs, which contributes to the eradication of the pathogens (19, 20, 53, 54).

To date, five classical cell-associated PRRs have been identified and characterized in several vertebrate species. These include three transmembrane PRRs—Toll-like receptors (TLRs), C-type lectin receptors (CLRs), and Class A scavenger receptors (cA-SRs)—and two cytoplasmic PRRs—RIG-I like receptors (RLRs) and NOD-like receptors (NLRs) (17, 20).

The TLR is a crucial class of PRRs; these transmembrane receptors play important roles in inflammation activation, IFN production, and shaping of adaptive immunity (20, 55). In mammals, the TLR family consists of up to 13 gene members. Ten TLRs were identified in human and 12 TLRs were identified in mouse, whereas at least 17 TLRs were detected in some fish species (20, 21, 56–61) and 11 to more than 20 TLRs in amphibians. For example, in the anuran *Bombina maxima*, a total of 11 *TLR* genes encoding 32 different transcripts have been reported, including gene orthologs of *TLR1*-*4*, *6*-*8* and *Toll*/*IL*-*1 receptor* (62). Twenty functional different *TLR* genes and several adaptor proteins were characterized in *Xenopus tropicalis* tadpoles and adults (22, 63, 64). While all the mammalian *TLR* gene orthologs exist in both *X. laevis* and *X. tropicalis*, some TLR family members such as TLR14 have been expanded in *Xenopu*s (13). In the Chinese giant salamander, 11 *AdTLRs* (*TLR 1*, *2*, *3*, *5*, *6*, *7*, *8*, *10*, *13*, *21*, *22*) have been identified. *AdTLR3*, *AdTLR5*, and *AdTLR7* share clear orthology with fish and mammalian counterparts. The bony fish-specific *TLR21* and *22* genes and the mouse-specific *TLR13* gene orthologs are present in *A. davidianus*, while *AdTLR2*, *AdTLR5*, and *AdTLR21* have duplicated gene copies (17). Genes encoding the downstream molecules of TLR signaling pathway including the adapter MyD88 and the pro-inflammatory cytokines such as TNFα, IL-1β, IFNγ, IL-6, and IL-18, have also been identified in *A. davidianus* (4, 17, 27). AdTLRs are less diversified than fish and *X. laevis* although some TLRs and the downstream pathways are conserved.

CLRs, the other transmembrane PRRs, are able to recognize carbohydrate structures of bacteria, fungi, parasites, and virus through a unique structure known as “C-type carbohydrate recognition domain (CRD)’’ or ‘‘C-type lectin domain” (65, 66). The CLR pathway initiates innate immune responses, including phagocytosis, activation of the complement system, and enhanced NK cell activity, and regulates adaptive immune responses (20, 65). Galectin-1, one important CLRs member, has been identified in *A. davidianus* (66). It contains a highly conserved CRD domain; it exhibits agglutination and binding activity to both Gram-positive and -negative bacteria as well as potential antiviral activity during GSIV infection (66). These limited results suggest diverse functions of CLRs in pathogen defense of *A. davidianus*.

cA-SR is a class of ancient transmembrane PRRs that recognize and bind low-density lipoproteins, bacteria and nucleic acids (61, 67). The cA-SR family includes five members: the scavenger receptor A (SR-A); the macrophage receptor with collagenous structure (MARCO); and the scavenger receptor A member 3 (SCARA3), SCARA4, and SCARA5. A conserved scavenger receptor cysteine-rich (SRCR) domain is present in SR-A, MARCO, and SCARA5, but not in SCARA3 and SCARA4 (61). Some cA-SRs serve as cellular receptors for DNA virus (67). In mammals, herpes simplex virus 1 (HSV-1) and vaccinia virus (VACV) use MARCO for cellular entry, and adenovirus 5 (AdV-5) uses both MARCO and SR-A. In amphibians, it has been reported that FV3 can enter cells *in vitro* by using cA-SRs (67). In *A. davidianus*, further studies are needed to characterize the roles of cA-SRs.

The RLR family members, which recognize viral RNA and induce IPS-1/VISA/Cardif (MAVS) signal and pro-inflammatory cytokines, play crucial roles in intracellular pattern recognition (20, 68). Three important RLR family members are present in *A. davidianus* as in other vertebrates, including retinoic acid inducible gene I (RIG-I), laboratory of genetics and physiology 2 (LGP2), and melanoma differentiation-gene 5 (MDA5). Among them, the AdRIG-I and AdMAD5 amino acid sequences contain three conserved elements: a N-terminal CARD domain, a DExD/H box RNA helicase domain, and a C-terminal RD domain. However, a CARD-containing adapter molecule *IPS-1* gene has not been identified in *A. davidianus* by transcriptomic sequencing and may be missing (17, 23). Although LGP2 lacks CARD domain in other vertebrate (69), the structure signature of AdLGP2 has not been investigated. The RLR activation pathway induces IFN regulatory factor 3 (IRF3) regulated type-I interferon response and subsequently stimulates the production of IFN-stimulated genes (*ISGs*) including *ISG15* and the *Myxovirus resistance* (*Mx*) gene, which has been characterized in the Chinese giant salamander (28, 69–71). Further characterization of the RLR pathway in *A. davidianus* should now be undertaken and in particular the absence of adapter IPS-1 will have to be confirmed.

NLRs, another important PRRs family, are intracellular sensors of pathogenic products that are able to induce innate pro-inflammatory responses (57). The NLR family is composed of three distinct subfamilies: NODs, NLRPs, and IPAF (72). Genes of these three subfamilies are present in the two *Xenopus* genomes. In *A. davidianus*, genes encoding the canonical NOD proteins (NOD1-3, NOD5, and CIITA), NAIP, IPAF, and apoptotic protease activating factor 1 (APAF1) were identified. Although NLRs did not show a major expansion, multiple isoforms of NOD2, NOD3, NALP3/NALP12, APAF1, IPAF, and CIITA were identified in *A. davidianus* by transcriptomic analysis (17). These isoforms suggest a complex and diversified detection system resulting from alternative splicing and gene duplication (17).

As soluble PRRs, the vertebrate complement system not only plays a critical role in potentiating humoral immune responses and in bridging the host innate and adaptive immune response, but also facilitates the ability of antibodies to clear pathogens. The complement system, including over 30 soluble plasma proteins and receptors, can be activated by three pathways: the classical pathway, the alternative pathway, and the lectin-mediated pathway to initiate defense against multiple pathogens (19, 20, 61). The activation of the classical pathway is triggered by bacterial lipopolysaccharide, pentraxins such as C-reactive protein (CRP), serum amyloid, etc. The alternative pathway is induced spontaneously and primarily depends on recognition of PAMPs. The lectin-mediated pathway is initiated by binding carbohydrate structures such as mannan-binding lectin (MBL), MBL-associated serine proteases (MASPs), and ficolins (53, 61). All these pathways converge to effector function including four sequential steps: (1) opsonization of bacteria for enhanced phagocytosis; (2) recruitment and activation of leukocytes at the site of inflammation; (3) generation of membrane-attack complexes to punch holes in the cell membranes of pathogens; and (4) pathogen destruction (20). Genes encoding proteins of these three pathways, such as C1-9 and MASP, have been identified in *X. laevis*, *X. tropicalis*, and *A. davidianus* (17, 73). Thus, according to recent data, the complement system is likely conserved between anura and urodele.

Another important type of immune effector molecule in amphibian is constituted by antimicrobial peptides (AMPs). The amphibian skin granular glands constitutively secrete AMPs that serve as a nonspecific physical and chemical barrier against a wide range of pathogens (24, 74). AMPs have also been reported active against ranavirus (75), although their protective activity is minimal for Eastern Hellbenders (76). Over 600 AMPs that belong to over 30 families were characterized in amphibians (24, 74, 77). Among those, Cathelicidin is a prominent family of AMPs that acts as multifunctional effector molecules in innate immunity. Cathelicidin is produced as a cathelicidin-like precursor. The critical cleavage step into mature cathelicidin is mediated by an elastase that recognizes the amino acid motive glycine-alanine-serine-threonine-isoleucine-valine. In the Chinese giant salamander, cathelicidin-like precursor transcripts are abundant in the skin (24).

### Adaptive Immune System

As a specific defense system, the adaptive immune system includes humoral immune responses with secreted antibodies and cellular immune responses with activated effector lymphocytes to eliminate specific pathogens after infection. B and T cells are the major cellular components in humoral immune responses and cellular immune responses, respectively. B and T cells expressed surface Ag-specific receptors that have undergone recombination-activating genes-dependent somatic recombination during their development (19).

T-cell receptors (TCRs) are essential for activation of cellular immune responses. After presented and complexed with the major histocompatibility complex (MHC) molecules, the pathogen-derived peptides are recognized by TCRs (19). MHC molecules, including MHC class I and MHC class II, are cell surface receptors. These two classes of receptors have different tertiary structures and expression patterns. Moreover, they interact with TCRs expressed by different types of T lymphocytes. The majority of cells of an organism express MHC class I molecules, which are heterodimers consisting of two subunits: the α subunit encoded in the MHC locus as a polymorphic MHC gene and the β subunit (β-2 microglobulin, β-2m) encoded by a non-polymorphic gene outside the MHC locus. The transmembrane α subunit binds to antigenic peptides that it presents at the cell surface to CD8 cytotoxic T cells. MHC class II molecules are heterodimeric α and β receptors encoded by polymorphic genes that are expressed by antigen-presenting cells (APCs) and that present antigenic peptides to CD4 T helper cells (19, 20).

Between mammals and amphibians, the structure and complexity of MHC molecules are similar. Moreover, the close correlation of the diversity of MHC alleles to disease resistance has been revealed (29, 53, 78). In anurans, depending on species, from one (*Xenopus*) to 2 or 3 highly polymorphic MHC class I genes per haplotype have been found (30, 79). In addition, in *Xenopus*, an extended family of oligomorphic *MHC class I-like* genes has been characterized outside the MHC locus (80). The existence of such *class I-like* genes in other anuran species remains to be determined. The number of *MHC class II a* and *β* genes varies between 1 and 2 among anuran species (19, 30–32). In the Chinese giant salamander, although the precise number of *MHC class I* and *class II* genes per genome is still unknown, a previous report has identified 26 *class IA*, 27 *class IIA*, and 17 *class IIB AdMHC* alleles and most variations between alleles have been detected in putative peptide-binding region (PBR) (29). Following GSIV infection, the transcription of *MHC* isoforms was increased, consistent with their roles in the immune response (29).

B-cell receptors (BCRs) are pivotal elements in humoral immune response that are expressed in two forms: (1) membrane receptors on B cell surface that recognize antigen, and (2) secreted immunoglobulins (Igs) present in the blood circulation and are produced in large amounts by plasma cells (20). As in mammals, genes encoding the BCRs and Igs in amphibians share conserved structure with a constant and variable domain in both light and heavy chains. Five Ig heavy chains have been characterized in *Xenopus*: IgM, IgY, IgX, IgD, and IgF (13, 19, 35–37, 81). IgM-positive cells are located in most host tissues, including spleen, liver, kidney, and gut. IgX-positive cells are mainly distributed at the epithelial layer of the gut and skin, and are less frequent in the spleen and liver, which suggests that IgX may be involved in mucosal immunity (82). *Xenopus* IgY-positive cells are located in the blood circulation, spleen, and liver but generally absent in the gut. IgY is the functional analog to mammalian IgG. The switch from IgM to IgY is thymus-dependent and temperature-dependent, and requires the collaboration of T cells (81). *Xenopus* IgD and IgF were not identified until 1996, and both of them are expressed mainly in the spleen. *Xenopus* IgD, which is orthologous to the isotype IgW of lungfish and IgW/X of cartilaginous fish, is considered as ancient as IgM. IgF consisting of two constant domains and a hinge region is the shortest Igs in *Xenopus*. The two constant domains are similar to those of fish and the fourth constant domains of IgY, which suggests that IgF is produced by tandem duplication of IgY followed by a loss of internal constant domains (19, 38, 82, 83).

From recent reports, genes encoding three IgH chains (AdIgD, AdIgM, and AdIgY) have been detected and characterized in *A. davidianus* (39). AdIgD displays four constant domains (C_H_1–C_H_4) and a hinge region, which is distinct from other amphibians. Furthermore, AdIgY△Fc, a unique IgY form that has not been described in other amphibians, was detected in serum of adult Chinese giant salamanders. Further diversification of AdIgH appears to occur by alternative splicing (39). Furthermore, *AdIgY* transcripts were detected by *in situ* hybridization in the thymus, kidney, liver, and spleen of *A. davidianus* (40). More recently, we have identified some Ig light chain transcripts by transcriptomic analysis, including *lambda light chain*, *light chain type III*, and *kappa light chain*. Their antiviral functions remain to be further studied.

## Lymphocyte Development and Homing

**Figure 1:**
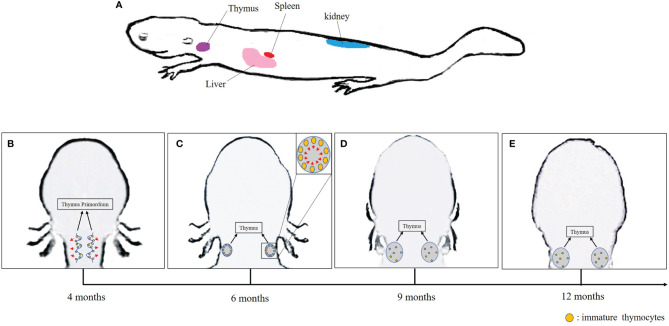
**(A)** Lymphopoietic tissues in Chinese giant salamander. **(B–E)** Schematic overview of the thymus development in the Chinese giant salamander. Arrows indicate the direction of migration of immature thymocytes.

It is well known that mammalian equivalent of lymph nodes are absent in *Xenopus*. In addition, the bone marrow has only a minimal role in hematopoiesis, which occurs mainly in the liver and the spleen. Thus, the spleen serves as both a primary lymphoid organ especially for B-cell differentiation, and a secondary lymphoid organ where B and T cells accumulate. As in mammals, the thymus is the site of T-cell differentiation and maturation in *Xenopus*. The liver and kidney are also sites of lymphocytes and other leukocyte accumulation (19, 53, 81, 84, 85). In *A. davidianus*, in addition to the thymus, spleen, and liver, the kidney also appears to serve as a lymphopoietic organ (**Figure 1A**) (40).

### T-Cell Differentiation

In jawed vertebrates, the differentiation and maturation of T lymphocyte primarily occur in the thymus (86). In gnathostomes, histogenesis of thymus has been described in four consecutive steps: (1) appearance of the thymus primordium; (2) colonization of the primordium by lymphocyte precursors, (3) initiation of thymocytes differentiation and expansion; and (4) formation of cortex and medulla architecture (87). In *Xenopus*, the thymus primordium emerges at 3 days post-fertilization (dpf), discernable cortex and medulla regions appear at 6–8 dpf, while lymphocyte precursors colonization followed by thymocytes differentiation occurs at 5–7 dpf onward (88–90). During metamorphosis, the majority of tadpole thymocytes die, and the thymus is colonized by new stem cells that differentiate into adult type thymocytes and mature T cells distinct from larval T cells (15, 88). In urodeles that undergo incomplete metamorphosis, the lymphoid precursor cells colonize the thymic pharyngeal buds at 3–4 weeks; they then spread and distribute in the adjacent mesenchyme before migrating to the thymus epithelial primordium. The immature thymocytes accumulate under the capsula and develop in a centripetal manner (16).

As expected, the thymus is the main site for T-cell differentiation in the Chinese giant salamander as in other jawed vertebrates. Significant expression of *rag1* and *rag2* indicative of TCR rearrangements in immature thymocytes were detected in thymus primordium in 4-month-old salamanders, which is similar to other salamanders, whose B- and T-cell differentiate from 2 months to 8 months after fertilization (14, 40). In *Xenopus*, a transitory pause of *rag* gene expression occurs during metamorphosis, which might be correlated with tadpole thymocyte deletion to make room for new adult thymocyte generation (90). More research is required to determine whether any thymocyte deletion might happen in *A. davidianus* from hatching stage approximately 42 dpf to 4 months of age (91). Interestingly, along with the thymocyte differentiation, the spatial distribution of *rag1*, *rag2*, and *tcrβ* signals changes over time in *A. davidianus*. Both *rag* and *tcrβ* transcripts were observed in the marginal area of the thymus primordium of 4-month-old salamanders. These transcripts became predominantly distributed in the sub-capsular region over the medulla in 6-month-old salamanders, and disseminated over the cortex and medulla of thymus after 9 months of age (**Figures 1B–E**). Although the expressions of *rag1* and *tcrβ* were definitely detected in the kidney, liver, and spleen, the roles of these organs in T-cell differentiation and homing remain to be determined (40). It is known, for example, that mature thymic-derived T cells migrate to *Xenopus* spleen and zebrafish kidney through blood circulation where they remain stored (19, 92). Therefore, we speculate that immature T cells differentiate in thymus and then mature T cells immigrate and remain stored in the kidney, liver, and spleen of the *A. davidianus* (40).

### B-Cell Differentiation

In contrast to mammals, the bone marrow is not the site for B-cell lymphopoiesis in most lower vertebrates. Instead, B-cell differentiation occurs in different tissues across jawed vertebrate species. The anterior kidney is the site for B-cell differentiation in most bony fish, whereas this is the spleen in urodels (90, 93, 94). In addition, the liver appears to serve as a transitory site of lymphopoietis in the Mexican axolotl (94). Both the liver and the spleen are considered to serve as sites of B-cell differentiation in *Xenopus* (19).

In the Chinese giant salamander, the spleen, kidney, and liver were identified as likely sites of B-cell differentiation. Notably, apparent lymphocyte differentiation was localized by *in situ* hybridization in adjacent zone of the blood vessel and the hematopoietic layer of the liver, and also at the periphery layer of the kidney (40). It is possible that the Chinese giant salamanders exhibit more sites of B-cell differentiation due to their evolutionary status between aquatic and terrestrial animals. As such, virus induces B-cell responses in Chinese giant salamanders may occur in multiple tissues (95). As an organized lymphoid structure, the *Xenopus* spleen is well delimited into a white pulp and red pulp (96). The white pulp has a lymphoid structure that is organized by concentric layers of lymphocytes surrounding a central artery (85). The white pulp is surrounded by red pulp that contains leukocytes and erythrocytes (97). In contrast to anuran amphibians, the red pulp and the white pulp are not well delimited in *A. davidianus* spleen (40).

## Antiviral Response to GSIV

GSIV infection of the Chinese giant salamander results in extremely high mortality (close to 100%) in both juvenile and adult salamanders. Infected salamanders show swollen head and neck, necrotic limbs, and hemorrhages on the skin (3, 6). Similar high mortality has been reported for ATV infection of larvae and adult tiger salamanders as well as for FV3 infection of *Xenopus* tadpoles (98, 99). During infection, transcript and protein levels of the GSIV major capsid protein (MCP) mainly increase in the kidney and spleen, which suggests that these organs are the major targets for GSIV replication (4). Similarly, the kidney is the main site of FV3 replication in adult frogs (13, 98). To determine whether the high susceptibility of *A. davidianus* to GSIV infection is due to an inefficient immune system, innate and adaptive immune responses need to be examined in more detail. We review below what has been undertaken to date.

### Innate Immune Response to GSIV

To assess the innate immune response of *A. davidianus* against GSIV, changes in relative expression of several innate immune genes in adult animals were monitored during infection by quantitative real-time PCR. As a critical element in intracellular pathogen recognition, *TLR7* gene expression was induced in the kidney and liver as early as 6 h following infection with GSIV and at 48 h post-infection (hpi) in the spleen (21). Other PRRs, such as the CLRs and RLRs, were also induced following GSIV infection. Indeed, expression of *AdGalectin-1* was increased at 1 day post-infection (dpi) in the kidney and both *RIG-1* and *MDA5* transcript levels were increased as early as 12 hpi in the kidney and spleen (23, 66). Thus, the *A. davidianus* host innate immune system is able to detect and recognize viral infection. Changes in expression of downstream genes of the RLR pathway, such as *IRF3*, *type I IFN*, and *ISGs* genes during GSIV infection were also monitored. *IRF3* expression was increased in the spleen at 3 hpi (28). *Type I IFN* transcript levels were upregulated in peripheral blood leukocytes at 12 hpi, while *Mx* transcripts levels were upregulated in the spleen at 6 hpi and in the kidney at 12 hpi (70, 71). Inflammatory genes such as *TNF-α* and *IL-1β* were significantly induced in the spleen at 3 dpi and in the kidney at 7 dpi, while the macrophage migration inhibitor (*mif*) gene was induced in the spleen at 3 dpi and in the kidney at 12 dpi. Collectively, these results suggest a faster and stronger inflammatory response in the spleen compared to the kidney following GSIV infection in the Chinese giant salamander (4). In adult *Xenopus*, FV3 infection also induces a rapid response (as early as 1dpi) of *type I IFN* and *Mx1* and *2*, as well as pro-inflammatory and inflammatory-related genes (*IFNγ*, *IL-1β*, and *TNF-α*) in the kidney (100, 101). In contrast, delayed and lower transcription of *IL-1β*, *TNFα*, and *IFNγ* genes was observed in FV3-infected tadpoles compared to adult frogs, and *type I* and *type III IFN* gene responses of tadpoles were distinct from adult frogs (100, 102–104). Overall, these results suggest that the innate immune response is efficiently triggered upon RV infection in the giant salamander as in amphibians and that there is no obvious defect of detection and defense by the innate immune system.

The genomic structure of *type I IFN* genes is different between higher and lower vertebrates. In mammals, birds and reptiles, *type I IFNs* genes are intron-less and are clustered in the same chromosome, whereas bony fish *type I IFNs* genes typically contain introns. *Xenopus* present an interesting intermediary state in the evolution of *IFN* genes by having expended families of both intron-less and intron-containing type I *IFN* genes (105). The Chinese giant salamander *type I IFNs* appear to consist of only intron-containing genes, which would be consistent with their ancient evolutionary origin antecedent to that of anuran amphibians such as *Xenopus*.

To further determine the ability of type I IFN to provide resistance to GSIV infection, recombinant Chinese giant salamander type I IFN and Mx1 were assayed *in vitro*. Transfected overexpressions of *type I IFN* and *Mx* genes in Chinese giant salamander cell lines led to inhibition of GSIV replication, which demonstrates their antiviral activity against GSIV (70, 71). Additionally, *IFNβ* and *ISGs* (*ISG15* and *Mx* genes) transcript levels were all decreased in *AdIRF3*-silenced leukocytes, which implicated AdIRF3 in the regulation of IFN signaling and ISG production (28). Using *in situ* hybridization, significantly less *MCP* transcripts were detected in the spleen from 7 dpi to 15 dpi, which suggested that high expressions of *TNF-α*, *IL-1β*, and *mif* genes might help to reduce GSIV replication (4). In adult *Xenopus*, FV3 clearance typically occurs within 2 weeks post-infection (106). Macrophages and NK cells are critical innate cell effectors in *Xenopus* host response during early stages of FV3 infection, and increased expression of *IL-1β*, *TNF-α*, *Mx1*, and *IFN-γ* genes was detected in infected tissues and leukocytes of adult frogs (13, 100, 107). These data reveal that the innate immune system functionally and critically contributes to reduce RVs replication in amphibians. However, RVs are able to overcome this initial innate host defense both in the Chinese giant salamander and *X. laevis* tadpoles.

Another innate effector system that may play a significant role in RVs defense is AMP secretion. In *Rana catesbeiana* and *Rana pipiens*, some AMPs can inactivate FV3 by direct contact or increase resistance to infection by enhancing immune responses (24, 108). In tiger salamanders, ATV-induced viral plaques in cell culture were reduced by some mixtures of AMPs, but the effects were different among these preparations (109). Skin AMPs from Hellbender also have detectable *in vitro* antiviral activity, albeit insufficient to provide effective protection to RVs (76). Five putative mature cathelicidins, which belong to an important family of AMPs, are predicted from transcriptomic data in the Chinese giant salamander skin, but their antiviral role remains to be investigated (24).

### Adaptive Immune Response to GSIV

The critical roles of MHC class I antigen presentation and activation of antiviral cytotoxic CD8 T cells have been rigorously demonstrated in *X. laevis* (13, 110). Likewise, the suboptimal function of classical MHC class I presentation in tadpoles until metamorphosis may explain in part the higher susceptibility of *X. laevis* tadpoles to FV3 (88, 100).

As mentioned above, 70 MHC alleles and multiple novel splice variants have been characterized in *A. davidianus*. Notably, full-length transcripts and individual splice variant isoforms of MHC class IA, class IIA, and class IIB have been found to be upregulated after GSIV infection, consistent with crucial roles of both MHC class I and class II molecules in host antiviral responses (29).

T- and B-lymphocyte responses correlate with viral clearance in adult *X. laevis* (13). The virus susceptibility of adult *X. laevis* was significantly increased after depletion of CD8 T cells, demonstrating the crucial role of CD8 T cells in anti-FV3 response (110). Upon primary ranaviral infection, the proliferation of CD8 T cells in the spleen and accumulation in the kidney appeared from day 6 onward, which was associated with FV3 clearance. During a secondary FV3 infection, earlier but lower proliferation and infiltration of CD8 T cell in parallel with faster clearance of FV3 was detected compared to the primary infection. These data provide evidence that CD8 T-cell memory and protective response are both involved in *X. laevis* immune defenses against FV3 (13, 110). According to microarray technology, ATV-infected axolotl showed upregulation of innate immune-related genes rather than adaptive immune-related genes (111). Consistent with the study in axolotl, expression levels of *CD8alpha* and *CD8beta* genes were downregulated in GSIV-infected Chinese giant salamander, suggesting a weak or inhibited CD8 T-cell response (26). However, more evidence is needed to establish whether the CD8 response is overcome, counteracted by GSIV, or inherently weak. Moreover, in *X. laevis*, an invariant T (iT) cell subset (iVα6), which is restricted by the MHC class I-like molecule XNC10 and expresses the invariant rearrangement Vα6-Ja1.43, is also critically involved in antiviral responses (101, 112, 113). In tadpoles, abrogation of Vα6 iT cell development significantly increased viral replication and host mortality at early stage of FV3 infection (101, 112). In adult *Xenopus*, the lack of Vα6 iT cells resulted in a less effective antiviral response, such as delayed viral clearance and more FV3-induce kidney damage (113). In addition, transitory depletion of Vα6 iT cells resulted in delayed interferon and cytokine genes responses and long-lasting negative inability to control FV3 infection in tadpoles, which further supported the important role of Vα6 iT cells in *Xenopus* immune surveillance system (114). Although iT cells have been demonstrated in *Xenopus* and mammals (112, 115), they are still unknown in *A. davidianus*.

While viral clearance may not be as efficient as *Ambystoma mexicanum* (4, 39), studies to date have not revealed an obvious lack of antiviral innate and adaptive immune response in *A. davidianus*. As such, it would be premature to conclude that the salamander immune system is deficient. Indeed, elicitation of effective and durable protective immunity has been obtained by vaccination in *A. davidianus* (93, 116, 117). Priming giant salamanders with self-assembly of the major capsid produced in the yeast expression system into virus-like particles induced long-lasting neutralizing antibodies and resulted in more than 50% protection of these animals to GSIV infection. These important findings imply that the high susceptibility of the Chinese giant salamander to GSIV is not due to intrinsic weakness of its immune system but perhaps rather the result of the recent spill out and transmission of a virulent ranavirus, that is able to overcome the host immune responses.

### Immune Evasion Strategies of RVs

Although evidence of immune evasion of GSIV is lacking, RV immune evasion strategies have been explored. Immune evasion genes are usually defined as genes that impair host antiviral responses and facilitate virus proliferation. Candidate immune evasion genes in RVs include the α subunit of eukaryotic initiation factor eIF-2 (vIF-2α), the caspase recruitment domain-containing protein (vCARD), hydroxysteroid dehydrogenase homolog (vHSD), the tumor necrosis factor receptor (vTNFR), the RNase III-like protein, and cytosine DNA methyltransferase (vDMT) (13). By analogy to a similar immune evasion in vaccinia virus, vIF-2α can partially inhibit phosphorylation of host eIF-2α by competitive binding to the antiviral protein kinase PKR and thus interfering with the host cell protein synthesis. vHSD is postulated to trigger glucocorticoid synthesis and enhance virus replication through suppression of the immune responses. vCARD may bind RIG-I/MDA5 and/or MAVS/ISP-1 followed by inhibition of interferon induction and/or apoptosis. The RNAse III-like protein has been postulated to block siRNA-mediated interference or to process viral mRNAs. The viral RNAse III can bind and/or degrade dsRNA and block the activation of PKR (118). Disruption of the eIF-2α gene in ATV resulted in increasing pathogenicity and sensitivity to interferon of the recombinant virus, indicating that *vIF-2α* gene is likely immune evasion gene (119). Interestingly, the disruption of the truncated vIF-2α in FV3 also led to reduced viral replication, increased survival and apoptosis, and higher sensitivity to IFN treatment, which suggests that in addition to blocking the activation of PKR, *vIF-2α* contributes to RV immune evasion through other unknown mechanisms (120, 121). Deletion of vCARD from FV3 resulted in low levels of viral replication as well as higher level of apoptosis and cell mortality *in vitro*, which suggested that vCARD might participate in the regulation of apoptosis. Furthermore, replication of vCARD mutant FV3 was affected in *Xenopus*-IFN treated cells, indicating that vCARD might contribute to interfering IFN response (121). HSD mutant FV3 also resulted in reduced viral replication and mortality but did not lead to a higher level of apoptosis, suggesting that vHSD contributes to viral pathogenesis (121). An additional immediate-early gene, 18K, of unknown function but conserved among RVs, is potentially involved in virulence. Compared to wild type, recombinant FV3 mutants with 18K deletion were found to induce more apoptosis and to replicate less, but also to be more resistant to r*Xl*IFN inhibition, which suggests that 18K that may contribute to virulence and even immune evasion by regulating timely FV3 gene expression and release (121). Only few GSIV viral genes have been investigated to date and their roles of immune evasion are still unclear. Overexpression of GSIV *13R* gene that encodes a viral non-structural protein containing a transmembrane domain (TMD) and a restriction endonuclease-like domain did not affect viral replication (122). In contrast, overexpression of GSIV *1R* gene that encodes virus late transcription factor-3 like (VLTF3 like) domain induced viral replication and promoted cell proliferation (123). Functional studies of GSIV genes and gene products are urgently needed not only for a better characterization of GSIV immune evasion strategies, but also for a broader understanding of *A. davidianus* immune responses.

## Conclusion and Perspectives

Although frogs and salamanders have colonized similar aquatic environment, the morphogenesis of their lymphopoietic tissues is distinct and it is likely that their immune systems exhibit differences too. However, in contrast with earlier studies implying a weak or even deficient immunity in salamanders, recent genomic and transcriptomic studies indicate that all the critical genetic elements of the innate and adaptive immune system are present in salamanders, suggesting that salamanders are not inherently immune defective. This is underscored by host responses to RV pathogens. Although salamanders are highly susceptible to RV infection, the fact that effective vaccination can be obtained in the giant salamanders demonstrates their immune competence, and rather implies that virulence and immune evasion factors allow GSIV pathogens to overcome their immune defenses. Thus, to advance both fundamental and applied immunology of salamanders and especially the giant salamander, it is important in our view, to integrate new genomic and transcriptomic technology with functional studies in the context of current infectious diseases caused by ranaviruses.

To date, major progress of recent studies in Chinese giant salamander immunology have been mainly focused on molecular identifications, while functional exploration has just begun. Useful immune gene sequence information has been obtained by high-throughput deep sequencing technologies, but due to a lack of genome sequences, antibodies, and reverse genetics applied to GSIV and salamanders, gene function often remains speculative compared to other amphibian models such as *Xenopus*. From our perspective, further research on Chinese giant salamander including better resources and tools must be implemented to increase a full understanding of the variation, adaptation, and plasticity of the immune system in jaw vertebrates as well as to achieve a better control of infectious disease caused be ranavirus worldwide. Among critical gap of knowledge imperative to fill, we have highlighted in this review the following: (1) when and where B lymphocyte and antibody secreting B cell develop and reside; (2) which immune cells present viral antigens; (3) importance of CD8 T-cell responses against ranavirus; (4) whether iT cells represent an important fraction of T cells like in *Xenopus* tadpoles and are critical in host defense against ranavirus; and (5) mechanisms of viral immune evasion.

In conclusion, future research on immune system of the giant salamander will not only contribute to understand the evolution of vertebrate immune systems, but also provide defensive strategy against virus disease in artificial culture.

## Author Contributions

NJ contributed ideas and drafted the first full version of the manuscript. JR and LZ revised and edited a later version of the manuscript. All authors contributed to the article and approved the submitted version.

## Funding

The work was supported by the National Natural Science Foundation of China (Grant No. 31702033), the Special Fund for Agro-Scientific Research in the Public Interest (2020XT0401), and the Hubei Provincial Natural Science Foundation of China (2020CFB347), as well as the IOS-1456213 from the National Science Foundation and R24AI059830 from the National Institute of Allergy and Infectious Diseases (NIH/NIAID) of USA.

## Conflict of Interest

The authors declare that the research was conducted in the absence of any commercial or financial relationships that could be construed as a potential conflict of interest.

## Publisher’s Note

All claims expressed in this article are solely those of the authors and do not necessarily represent those of their affiliated organizations, or those of the publisher, the editors and the reviewers. Any product that may be evaluated in this article, or claim that may be made by its manufacturer, is not guaranteed or endorsed by the publisher.

## References

[1] Pyron RA Wiens JJ. A Large-Scale Phylogeny of Amphibia Including Over 2800 Species, and a Revised Classification of Extant Frogs, Salamanders, and Caecilians. Mol Phylogenet Evol (2011) 61:543–83. doi: 10.1016/j.ympev.2011.06.012 21723399

[2] Zhang P Chen YQ Liu YF Zhou H Qu LH. The Complete Mitochondrial Genome of the Chinese Giant Salamander, *Andrias Davidianus* (Amphibia: Caudata). Gene (2003) 311:93–8. doi: 10.1016/S0378-1119(03)00560-2 12853142

[3] Meng Y Ma J Jiang N Zeng L Xiao H. Pathological and Microbiological Findings From Mortality of the Chinese Giant Salamander (*Andrias Davidianus*). Arch Virol (2014) 159:1403–12. doi: 10.1007/s00705-013-1962-6 24385158

[4] Jiang N Fan Y Zhou Y Liu W Ma J Meng Y. Characterization of Chinese Giant Salamander Iridovirus Tissue Tropism and Inflammatory Response After Infection. Dis Aquat Organisms (2015) 114:229–37. doi: 10.3354/dao02868 26036830

[5] Turvey ST Chen S Tapley B Liang Z Wei G Yang J. From Dirty to Delicacy? Changing Exploitation in China Threatens the World’s Largest Amphibians. People Nat (2021) 00:1–11. doi: 10.1002/pan3.10185

[6] Geng Y Wang KY Zhou ZY Li CW Wang J He M. First Report of a Ranavirus Associated With Morbidity and Mortality in Farmed Chinese Giant Salamanders (*Andrias Davidianus*). J Comp Pathol (2011) 145:95–102. doi: 10.1016/j.jcpa.2010.11.012 21256507

[7] Dong W Zhang X Yang C An J Qin J Song F. Iridovirus Infection in Chinese Giant Salamanders, China. Emerging Infect Dis (2011) 17:2388–9. doi: 10.3201/eid1712.101758 PMC3311219 22172343

[8] Chen ZY Gui JF Gao XC Pei C Hong YJ Zhang QY. Genome Architecture Changes and Major Gene Variations of *Andrias Davidianus* Ranavirus (ADRV). Vet Res (2013) 44:101. doi: 10.1186/1297-9716-44-101 24143877 PMC4015033

[9] Zhang QY Gui JF. Virus Genomes and Virus-Host Interactions in Aquaculture Animals. Sci China Life Sci (2015) 58:156–69. doi: 10.1007/s11427-015-4802-y 25591452

[10] Gui L Chinchar VG Zhang Q. Molecular Basis of Pathogenesis of Emerging Viruses Infecting Aquatic Animals. Aquacult Fisheries (2018) 3:1–5. doi: 10.1016/j.aaf.2017.12.003

[11] Gray MJ Miller DL Hoverman JT. Ecology and Pathology of Amphibian Ranaviruses. Dis Aquat Organisms (2009) 87:243–66. doi: 10.3354/dao02138 20099417

[12] Stöhr AC López-Bueno A Blahak S Caeiro MF Rosa GM de Matos APA. Phylogeny and Differentiation of Reptilian and Amphibian Ranaviruses Detected in Europe. PloS One (2015) 10:e0118633. doi: 10.1371/journal.pone.0118633 25706285 PMC4338083

[13] Chen G Robert J. Antiviral Immunity in Amphibians. Virus (2011) 3:2065–86. doi: 10.3390/v3112065 PMC3230842 22163335

[14] Godwin JW Rosenthal N. Scar-Free Wound Healing and Regeneration in Amphibians: Immunological Influence on Regenerative Success. Differentiation (2014) 87:66–75. doi: 10.1016/j.diff.2014.02.002 24565918

[15] Robert J Edholm E. A Prominent Role for Invariant T Cells in the Amphibian *Xenopus Laevis* Tadpoles. Immunogenetics (2014) 6:513–23. doi: 10.1007/s00251-014-0781-6 24898512

[16] Durand C Charlemagne J Fellah JS. Structure and Developmental Expression of Ikaros in the Mexican Axolot. Immunogenetics (1999) 50:336–43. doi: 10.1007/s002510050610 10630298

[17] Fan Y Chang MX Ma J Lapatra SE Hu YW Huang L. Transcriptomic Analysis of the Host Response to an Iridovirus Infection in Chinese Giant Salamander, Andrias Davidianus. Vet Res (2015) 46:136. doi: 10.1186/s13567-015-0279-8 26589400 PMC4654921

[18] Nowoshilow S Schloissnig S Fei J Dahl A Pang AWC Pippel M. The Axolotl Genome and the Evolution of Key Tissue Formation Regulators. Nature (2018) 554:50–5. doi: 10.1038/nature25458 29364872

[19] Robert J Ohta Y. Comparative and Developmental Study of the Immune System in *Xenopus* . Dev Dynamics (2009) 238:1249–70. doi: 10.1002/dvdy.21891 PMC2892269 19253402

[20] Zhu L Nie L Zhu G Xiang L Shao J. Advances in Research of Fish Immune-Relevant Genes: A Comparative Overview of Innate and Adaptive Immunity in Teleosts. Dev Comp Immunol (2013) 39:39–62. doi: 10.1016/j.dci.2012.04.001 22504163

[21] Huang L Fan Y Zhou Y Jiang N Liu W Meng Y. Cloning, Sequence Analysis and Expression Profiles of Toll-Like Receptor 7 From Chinese Giant Salamander Andrias Davidianus. Comp Biochem Physiol B- Biochem Mol Biol (2015) 184:52–7. doi: 10.1016/j.cbpb.2015.02.006 25754925

[22] Ishii A Kawasaki M Matsumoto M Tochinai S Seya T. Phylogenetic and Expression Analysis of Amphibian *Xenopus* Toll-Like Receptors. Immunogenetics (2007) 59:281–93. doi: 10.1007/s00251-007-0193-y 17265063

[23] Meng Y Fan Y Zhou Y Jiang N Xue M Liu W. Identification and Comparative Expression Analysis of *RIG-1* and *MDA5* in Chinese Giant Salamander Andrias Davidiaus. Aquat Res (2020) 00:1–8. doi: 10.1111/are.14803

[24] Meng Y Tian HF Hu QM Xiao HB. Molecular Cloning of Cathelicidin-Like cDNA From *Andrias Davidianus* . Russian J Genet (2018) 54:75–82. doi: 10.1134/S102279541801012X

[25] Matthijs S Ye L Stijlemans B Cornelis P Bossuyt F Roelants K. Low Structural Variation in the Host-Defence Peptide Repertoire of the Dwarf Clawed Frog *Hymenochirus Boettgeri* (Pipidae). PloS One (2014) 9:e86339. doi: 10.1371/journal.pone.0086339 24466037 PMC3899252

[26] Ke F Gui J Chen Z Li T Lei C Wang Z. Divergent Transcriptomic Responses Underlying the Ranaviruses-Amphibian Interaction Process on Interspecies Infection of Chinese Giant Salamander. BMC Genomics (2018) 19:211. doi: 10.1186/s12864-018-4596-y 29558886 PMC5861657

[27] Wang L Xu Y Zhou Y Liu Z Li B Gu W. The First Evidence of Four Transcripts From Two Interleukin 18 Genes in Animal and Their Involvement in Immune Responses in the Largest Amphibian Andrias davidiaus. Dev Comp Immunol (2020) 106:103598. doi: 10.1016/j.dci.2019.103598 31881236

[28] Xu Y Wang L Zhou Y Xiao Y Gu W Li B. Identification and Functional Analysis of Two Interferon Regulatory Factor 3 Genes and Their Involvement in Antiviral Immune Responses in the Chinese Giant Salamander Andrias Davidiaus. Dev Comp Immunol (2020) 110:103710. doi: 10.1016/j.dci.2020.103710 32311388

[29] Zhu R Chen Z Wang J Yuan J Liao X Gui J. Extensive Diversification of MHC in Chinese Giant Salamanders *Andrias Davidianus* (Anda-MHC) Reveals Novel Splice Variants. Dev Comp Immunol (2014) 42:311–22. doi: 10.1016/j.dci.2013.10.001 24135718

[30] Ohta Y Goetz W Hossain MZ Nonaka M Flajnik MF. Ancestral Organization of the MHC Revealed in the Amphibian. Xenopus J Immunol (2006) 176:3674–85. doi: 10.4049/jimmunol.176.6.3674 16517736

[31] Shum BP Avila D Du Pasquier L Kasahara M Flajnik MF. Isolation of a Classical MHC Class I cDNA From an Amphibian. Evidence for Only One Class I Locus in the *Xenopus* MHC. J Immunol (1993) 151:5376–86. 8228232

[32] Sato K Flajnik MF Du Pasquier L Katagiri M Kasahara M. Evolution of the MHC: Isolation of Class II Beta-Chain cDNA Clones From the Amphibian *Xenopus Laevis* . J Immunol (1993) 150:2831–43. 8454860

[33] Liu Y Kasahara M Rumfelt LL Flajnik MF. *Xenopus* Class II A Genes: Studies of Genetics, Polymorphism, and Expression. Dev Comp Immunol (2002) 26:735–50. doi: 10.1016/s0145-305x(02)00034-4 12206837

[34] Stewart R Ohta Y Minter RR Gibbons T Horton TL Ritchie P. Cloning and Characterization of *Xenopus* Beta2-Microglobulin. Dev Comp Immunol (2005) 29:723–32. doi: 10.1016/j.dci.2004.12.004 15854684

[35] Schwager J Mikoryak CA Steiner LA. Amino Acid Sequence of Heavy Chain From *Xenopus Laevis* IgM Deduced From cDNA Sequence: Implications for Evolution of Immunoglobulin Domains. Proc Natl Acad Sci USA (1988) 85:2245–9. doi: 10.1073/pnas.85.7.2245 PMC279967 2451244

[36] Lee A Hsu E. Isolation and Characterization of the *Xenopus* Terminal Deoxynucleotidyl Transferase. J Immunol (1994) 152:4500–7. 8157965

[37] Ohta Y Flajnik M. IgD, Like IgM, Is a Primordial Immunoglobulin Class Perpetuated in Most Jawed Vertebrates. Proc Natl Acad Sci USA (2006) 103:10723–8. doi: 10.1073/pnas.0601407103 PMC1636022 16818885

[38] Zhao Y Pan-Hammarström Q Yu S Wertz N Zhang X Li N. Identification of IgF, a Hinge-Region-Containing Ig Class, and IgD in Xenopus Tropicalis. Proc Natl Acad Sci USA (2006) 103:12087–92. doi: 10.1073/pnas.0600291103 PMC1567701 16877547

[39] Zhu R Chen Z Wang J Yuan J Liao X Gui J. Thymus cDNA Library Survey Uncovers Novel Features of Immune Molecules in Chinese Giant Salamander Andrias Davidianus. Dev Comp Immunol (2014) 46:413–22. doi: 10.1016/j.dci.2014.05.019 24909429

[40] Jiang N Fan Y Zhou Y Liu W Robert J Zeng L. *Rag1* and *Rag2* Gene Expressions Identify Lymphopoietic Tissues in Juvenile and Adult Chinese Giant Salamander (*Andrias Davidianus*). Dev Comp Immunol (2018) 87:24–35. doi: 10.1016/j.dci.2018.05.018 29800626

[41] Schwager J Bűrckert N Schwager M Wilson M. Evolution of Immunoglobulin Light Chain Genes: Analysis of *Xenopus* IgL Isotypes and Their Contribution to Antibody Diversity. EMBO J (1991) 10:505–11. doi: 10.1002/j.1460-2075.1991.tb07976.x PMC452677 1705882

[42] Qin T Ren L Hu X Guo Y Fei J Zhu Q. Genomic Organization of the Immunoglobulin Light Chain Gene Loci in *Xenopus Tropicalis*: Evolutionary Implications. Dev Comp Immunol (2008) 32:156– 165. doi: 10.1016/j.dci.2007.05.007 17624429

[43] Haire RN Shamblott MJ Amemiya CT Litman GW. A Second *Xenopus* Immunoglobulin Heavy Chain Constant Region Isotype Gene. Nucleic Acids Res (1989) 17:1776. doi: 10.1093/nar/17.4.1776 16617486 PMC331852

[44] Amemiya CT Haire RN Litman GW. Nucleotide Sequence of a cDNA Encoding a Third Distinct *Xenopus* Immunoglobulin Heavy Chain Isotype. Nucleic Acids Res (1989) 17:5388. doi: 10.1093/nar/17.13.5388 2503814 PMC318127

[45] Chretien I Marcuz A Fellah J Charlemagne J Du Pasquier L. The T Cell Receptor Beta Genes of *Xenopus* . Eur J Immunol (1997) 27:763–71. doi: 10.1002/eji.1830270327 9079820

[46] Haire RN Kitzan Haindfield MK Turpen JB Litman GW. Structure and Diversity of T-Lymphocyte Antigen Receptors Alpha and Gamma in. Xenopus Immunogenet (2002) 54:431–8. doi: 10.1007/s00251-002-0474-4 12242593

[47] Greenhalgh P Olesen CE Steiner LA. Characterization and Expression of Recombination Activating Genes (*RAG-1* and *RAG-2*) in *Xenopus Laevis* . J Immunol (1993) 151:3100–10. 8376769

[48] Ichikawa HT Sowden MP Torelli AT Bachl J Huang P Dance GS. Structural Phylogenetic Analysis of Activation-Induced Deaminase Function. J Immunol (2006) 177:355–61. doi: 10.4049/jimmunol.177.1.355 16785531

[49] Bernard D Hansen JD Du Pasquier L Lefranc MP Benmansour A Boudinot P. Costimulatory Receptors in Jawed Vertebrates: Conserved CD28, Odd CTLA4 and Multiple BTLAs. Dev Comp Immunol (2007) 31:255–71. doi: 10.1016/j.dci.2006.06.003 16928399

[50] Qi ZT Nie P. Comparative Study and Expression Analysis of the Interferon Gamma Gene Locus Cytokines in *Xenopus Tropicalis* . Immunogenetics (2008) 60:699–710. doi: 10.1007/s00251-008-0326-y 18726591

[51] Guselnikov SV Bell A Najakshin AM Robert J Taranin AV. Signaling FcRgamma and TCRzeta Subunit Homologs in the Amphibian *Xenopus Laevis* . Dev Comp Immunol (2003) 27:727–33. doi: 10.1016/s0145-305x(03)00055-7 12798368

[52] Gőbel TW Meier EL Du Pasquier L. Biochemical Analysis of the *Xenopus Laevis* TCR/CD3 Complex Supports the “Stepwise Evolution” Model. Eur J Immunol (2000) 30:2775–81. doi: 10.1002/1521-4141(200010)30:10<2775::AID-IMMU2775>3.0.CO;2-U 11069057

[53] Grogan LF Robert J Berger L Skerratt LF Scheele BC Castley JG. Review of the Amphibian Immune Response to Chytridiomycosis, and Future Directions. Front Immunol (2018) 9:2536. doi: 10.3389/fimmu.2018.02536 30473694 PMC6237969

[54] Grogan LF Humphries JE Robert J Lanctôt CM Nock CJ. Newell DA Et al. Immunological Aspects of Chytridiomycosis. J Fungi (Basel) (2020) 6:234. doi: 10.3390/jof6040234 PMC7712659 33086692

[55] Iwasaki A Medzhitov R. Toll-Like Receptor Control of the Adaptive Immune Responses. Nat Immunol (2004) 5:987–95. doi: 10.1038/ni1112 15454922

[56] Zhu R Du H Li S Li Y Ni H Yu X. *De Novo* Annotation of the Immune-Enriched Transcriptome Provides Insights Into Immune System Genes of Chinese Sturgeon (*Acipenser Sinensis*). Fish Shellfish Immunol (2016) 55:699–716. doi: 10.1016/j.fsi.2016.06.051 27368537

[57] Ariffin JK Sweet MJ. Differences in the Repertoire, Regulation and Function of Toll-Like Receptors and Inflammasome-Forming Nod-Like Receptors Between Human and Mouse. Curr Opin Microbiol (2013) 16:303–10. doi: 10.1016/j.mib.2013.03.002 23540353

[58] Quiniou SM Boudinot P Bengtén E. Comprehensive Survey and Genomic Characterization of Toll-Like Receptors (TLRs) in Channel Catfish, *Ictaluruspunctatus*: Identifcation of Novel Fish TLRs. Immunogenetics (2013) 65:511–30. doi: 10.1007/s00251-013-0694-9 23558557

[59] Kanwal Z Wiegertjes GF Veneman WJ Meijer AH Spaink HP. Comparative Studies of Toll-Like Receptor Signaling Using Zebrafish. Dev Comp Immunol (2014) 46:35–52. doi: 10.1016/j.dci.2014.02.003 24560981

[60] Pietretti D Wiegertjes GF. Ligand Specificities of Toll-Like Receptors in Fish: Indications From Infection Studies. Dev Comp Immunol (2014) 43:205–22. doi: 10.1016/j.dci.2013.08.010 23981328

[61] Jiang N Fan Y Zhou Y Wang W Ma J Zeng L. Transcriptome Analysis of *Aeromonas Hydrophila* Infected Hybrid Sturgeon (*Huso Dauricus*×*Acipenser Schrenckii*). Sci Rep (2018) 8:17925. doi: 10.1038/s41598-018-36376-2 30560883 PMC6298973

[62] Zhao F Yan C Wang X Yang Y Wang G Lee W. Comprehensive Transcriptome Profiling and Functional Analysis of the Frog (*Bombina Maxima*) Immune System. DNA Res (2014) 21:1–13. doi: 10.1093/dnares/dst035 23942912 PMC3925390

[63] Fitzgerald KA Palsson-McDermott EM Bowie AG Jefferies CA Mansell AS Brady G. Mal (MyD88-Adapter-Like) Is Required for Toll-Like Receptor-4 Signal Transduction. Nature (2001) 413:78. doi: 10.1038/35092578 11544529

[64] Roach JC Glusman G Rowen L Kaur A Purcell MK Smith KD. The Evolution of Vertebrate Toll-Like Receptors. Proc Natl Acad Sci USA (2005) 102:9577–82. doi: 10.1073/pnas.0502272102 PMC1172252 15976025

[65] Osorio F Reis e Sousa C. Myeloid C-Type Lectin Receptors in Pathogen Recognition and Host Defense. Immunity (2011) 34:651–64. doi: 10.1016/j.immuni.2011.05.001 21616435

[66] Yang H Lan Q Liu R Cui D Liu H Xiong D. Characterization of Galectin-1 From Chinese Giant Salamanders *Andrias Davidianus* and Its Involvements During Immune Response. Dev Comp Immunol (2017) 70:59–68. doi: 10.1016/j.dci.2017.01.004 28065604

[67] Vo N TK Guerreiro M Yaparla A Grayfer L J. DeWitte-Orr S. Class A Scavenger Receptors Are Used by Frog Virus 3 During Its Cellular Entry. Viruses (2019) 11:93. doi: 10.3390/v11020093 PMC6409810 30678064

[68] Yoneyama M Onomoto K Jogi M Akaboshi T Fujita T. Viral RNA Detection by RIG-I-Like Receptors. Curr Opin Immunol (2015) 32:48–53. doi: 10.1016/j.coi.2014.12.012 25594890

[69] Yoneyama M Fujita T. RNA Recognition and Signal Transduction by RIG-I-Like Receptors. Immunol Rev (2009) 227:54–65. doi: 10.1111/j.1600-065X.2008.00727.x 19120475

[70] Chen Q Ma J Fan Y Meng Y Xu J Zhou Y. Identification of Type I IFN in Chinese Giant Salamander (*Andrias Davidianus*) and the Response to an Iridovirus Infection. Mol Immunol (2015) 65:350–9. doi: 10.1016/j.molimm.2015.02.015 25733388

[71] Liu Y Li Y Zhou Y Nan J Fan Y Zeng L. Characterization, Expression Pattern and Antiviral Activities of *Mx* Gene in Chinese Giant Salamander, *Andrias Davidianus* . Int J Moleculars Sci (2020) 21:2246. doi: 10.3390/ijms21062246 PMC7139979 32213935

[72] Schroder K Tschopp J. The Inflammasomes. Cell (2010) 140:821–32. doi: 10.1016/j.cell.2010.01.040 20303873

[73] Fujita T Endo Y Nonaka M. Primitive Complement System - Recognition and Activation. Mol Immunol (2004) 41:103–11. doi: 10.1016/j.molimm.2004.03.026 15159055

[74] Rollins-Smith LA Conlon JM. Antimicrobial Peptide Defenses Against Chytridiomycosis an Emerging Infectious Disease of Amphibian Populations. Dev Comp Immunol (2005) 29:589–98. doi: 10.1016/j.dci.2004.11.004 15784290

[75] Chinchar VG Wang J Murti G Carey C Rollins- Smith L. Inactivation of Frog Virus 3 and Channel Catfish Virus by Esculentin-2p and Ranatuerin-2p, Two Antimicrobial Peptides Isolated From Frog Skin. Virology (2001) 288:351–7. doi: 10.1006/viro.2001.1080 11601906

[76] Cusaac JPW Cater ED Woodhams DC Robert J Spatz JA Howard JL. Emerging Pathogens and a Current-Use Pesticide: Potential Impacts on Eastern Hellbenders (UAAH-2019-0046). J Aquat Anim Health (2021) 33:24–32. doi: 10.1002/aah.10117 33590581

[77] Afshar M Gallo RL. Innate Immune Defense System of the Skin. Vet Dermatol (2013) 24:32–9. doi: 10.1111/j.1365-3164.2012.01082.x 23331677

[78] Teacher AGF Garner TWJ Nichols RA. Evidence for Directional Selection at a Novel Major Histocompatibility Class I Marker in Wild Common Frogs (*Rana Temporaria*) Exposed to a Viral Pathogen (*Ranavirus*). PloS One (2009) 4:e4616. doi: 10.1371/journal.pone.0004616 19240796 PMC2643007

[79] Piertney SB Oliver MK. The Evolutionary Ecology of the Major Histocompatibility Complex. Heredity (2006) 96:7–21. doi: 10.1038/sj.hdy.6800724 16094301

[80] Edholm ES Goyos A Taran J De Jesús Andino F Ohta Y Robert J. Unusual Evolutionary Conservation and Further Species-Specific Adaptations of a Large Family of Nonclassical MHC Class Ib Genes Across Different Degrees of Genome Ploidy in the Amphibian Subfamily. Xenopodinae Immunogenet (2014) 66:411–26. doi: 10.1007/s00251-014-0774-5 PMC4096976 24771209

[81] Du Pasquier L Robert J Courtet M Mussmann R. B-Cell Development in the Amphibian *Xenopus* . Immunol Rev (2000) 175:201–13. doi: 10.1111/j.1600-065x.2000.imr017501.x 10933604

[82] Mussmann R Du Pasquier L Hsu E. Is *Xenopus* IgX an Analog of IgA? Eur J Immunol (1996) 26:2823–30. doi: 10.1002/eji.1830261205 8977274

[83] Ota T Rast JP Litman GW Amemiya CT. Lineage-Restricted Retention of a Primitive Immunoglobulin Heavy Chain Isotype Within the *Dipnoi* Reveals an Evolutionary Paradox. Proc Natl Acad Sci USA (2003) 100:2501–6. doi: 10.1073/pnas.0538029100 PMC151370 12606718

[84] Marr S Morales H Bottaro A Cooper M Flajnik M Robert J. Localization and Differential Expression of Activation-Induced Cytidine Deaminase in the Amphibian *Xenopus* Upon Antigen Stimulation and During Early Development. J Immunol (2007) 179:6783–9. doi: 10.4049/jimmunol.179.10.6783 17982068

[85] Neely HR Guo J Flowers EM Criscitiello MF Flajnik M. “Double-Duty” Conventional Dendritic Cells in the Amphibian *Xenopus* as the Prototype for Antigen Presentation to B Cells. Eur J Immunol (2018) 48:430–40. doi: 10.1002/eji.201747260 PMC5844829 29235109

[86] Gordon J Manley NR. Mechanisms of Thymus Organogenesis and Morphogenesis. Development (2011) 138:3865–78. doi: 10.1242/dev.059998 PMC3160085 21862553

[87] Castillo A Razquin BE Lopez-Fierro P Alvarez F Zapata A Villena AJ. Enzyme- and Immuno-Histochemical Study of the Thymic Stroma in the Rainbow Trout, *Salmo Jairdneri* Richardson. Thymus (1990) 15:153–66. 1695032

[88] Flajnik MF Du Pasquier L. The Major Histocompatibility Complex of Frogs. Immunol Rev (1990) 113:47–63. doi: 10.1111/j.1600-065x.1990.tb00036.x 2180810

[89] Robert J Cohen N. Ontogeny of CTX Expression in *Xenopus* . Dev Comp Immunol (1998) 22:605–12. doi: 10.1016/s0145-305x(98)00028-7 9877440

[90] Hansen JD Zapata AG. Lymphocyte Development in Fish and Amphibians. Immunol Rev (1998) 166:199–220. doi: 10.1111/j.1600-065x.1998.tb01264.x 9914914

[91] Wang Y Chen H Wang F Rao F. Improved Method in Breeding and Artificial Propagation for Chinese Giant Salamander (*Andrias Davidianus*). J Marine Biol Aquacult (2017) 3:1–5. doi: 10.15436/2381-0750.17.1349

[92] Langenau DM Ferrando AA Traver D Kutok JL Hezel JD Kanki JP. *In Vivo* Tracking of T Cell Development, Ablation, and Engraftment in Transgenic Zebrafish. Proc Natl Acad Sci USA (2004) 101:7369–74. doi: 10.1073/pnas.0402248101 PMC409925 15123839

[93] Trede NS Zon LI. Development of T-Cell During Fish Embryogenesis. Dev Comp Immunol (1998) 22:253–63. doi: 10.1016/s0145-305x(98)00009-3 9700456

[94] Durand C Charlemagne J Fellah JS. RAG Expression is Restricted to the First Year of Life in Mexican Axoloti. Immunogenetics (2000) 51:681–7. doi: 10.1007/s002510000191 10941839

[95] Liu W Xu J Ma J LaPatra SE Meng Y Fan Y. Immunological Response and Protection in Chinese Giant Salamander *Andrias Davidianus* Immunized With Inactivated Iridovirus. Vet Microbiol (2014) 174:382–90. doi: 10.1016/j.vetmic.2014.10.028 25465180

[96] Manning MJ Horton JD. Histogenesis of Lymphoid Organs in Larvae of the South African Clawed Toad, *Xenopus Laevis* (Daudin). J Embryol Exp Morphol (1969) 22:265–77. 5361557

[97] Bricker NK Raskin RE Densmore CL. Cytochemical and Immunocytochemical Characterization of Blood Cells and Immunohistochemical Analysis of Spleen Cells From 2 Species of Frog, *Ran*a (*Aquarana*) *Catesbeiana* and *Xenopus Laevis* . Vet Clin Pathol (2012) 3:353–61. doi: 10.1111/j.1939-165X.2012.00452.x 22954297

[98] Gantress J Maniero GD Cohen N Robert J. Development and Characterization of a Modal System to Study Amphibian Immune Responses to Iridoviruses. Virology (2003) 311:254–62. doi: 10.1016/s0042-6822(03)00151-x 12842616

[99] Chinchar VG Hyatt A Miyazaki T Wiiiams T. Family Iridoviridae: Poor Viral Relation No Longer. Curr. Top. Microbiol. Immunology (2009) 328:123–70. doi: 10.1007/978-3-540-68618-7_4 19216437

[100] De Jesús Andino F Chen G Li Z Grayfer L Robert J. Susceptibility of *Xenopus Laevie* Tadpoles to Infection by the Ranavirus Frog-Virus 3 Correlates With a Reduced and Delayed Innate Immune Response in Comparison With Adult Frogs. Virology (2012) 432:435–43. doi: 10.1016/j.virol.2012.07.001 PMC3574294 22819836

[101] Robert J Eva-Stina E Sanchez J Torres-Luquis O De Jesús Andino F. *Xenopus-*FV3 Host-Pathogen Interactions and Immune Evasion. Virology (2017) 511:309–19. doi: 10.1016/j.virol.2017.06.005 PMC5623633 28625407

[102] Grayfer L De Jesús Andino F Robert J. Prominent Amphibian (*Xenopus Laevis*) Tadpole Type III Interferon Response to the Frog Virus 3 Ranavirus. J Virol (2015) 89:5072–82. doi: 10.1128/JVI.00051-15 PMC4403449 25717104

[103] Wendel ES Yaparla A Koubourli DV Grayfer L. Amphibian (*Xenopus Laevis*) Tadpoles and Adult Frogs Mount Distinct Interferon Responses to the Frog Virus 3 Ranavirus. Virology (2017) 503:12–20. doi: 10.1016/j.virol.2017.01.001 28081430

[104] Wendel ES Yaparla A Melnyk MLS Koubourli DV Grayfer L. Amphibian (*Xenopus Laevis*) Tadpoles and Adult Frogs Differ in Their Use of Expanded Repertoires of Type I and Type III Interferon Cytokines. Viruses (2018) 10:372. doi: 10.3390/v10070372 PMC6070924 30018186

[105] Sang Y Liu Q Lee J Ma W McVey DS Blecha F. Expansion of Amphibian Intronless Interferons Revises the Paradigm for Interferon Evolution and Functional Diversity. Sci Rep (2016) 6:29072. doi: 10.1038/srep29072 27356970 PMC4928184

[106] Robert J Morales H Buck W Cohen N Marr S Gantress J. Adaptive Immunity and Histopathology in Frog Virus 3-Infected. Xenopus Virol (2005) 332:667–75. doi: 10.1016/j.virol.2004.12.012 15680432

[107] Morales HD Abramowitz L Gertz J Sowa J Vogel A Robert J. Innate Immune Responses and Permissiveness to Ranavirus Infection of Peritoneal Leukocytes in the Frog *Xenopus Laevis* . J Virol (2010) 84:4912–22. doi: 10.1128/JVI.02486-09 PMC2863837 20200236

[108] Chinchar VG Bryan L Silphadaung U Noga E Wade D Rollins- Smith L. Inactivation of Viruses Infecting Ectothermic Animals by Amphibian and Piscine Antimicrobial Peptides. Virology (2004) 323:268–75. doi: 10.1016/j.virol.2004.02.029 15193922

[109] Sheafor B Davidson EW Parr L Rollins-Smith L. Antimicrobial Peptide Defenses in the Salamander, *Ambystoma Tigrinum*, Against Emerging Amphibian Pathogens. J Wildlife Dis (2008) 44:226–36. doi: 10.7589/0090-3558-44.2.226 18436656

[110] Morales HD Robert J. Characterization of Primary and Memory CD8 T-Cell Responses Against Ranavirus (FV3) in *Xenopus Laevis* . J Virol (2007) 81:2240–8. doi: 10.1128/JVI.01104-06 PMC1865961 17182687

[111] Cotter JD Storfer A Page RB Beachy CK Voss SR. Transcriptional Response of Mexican Axolotls to *Ambystoma Tigrinum Virus* (ATV) Infection. BMC Genomics (2008) 9:493. doi: 10.1186/1471-2164-9-493 18937860 PMC2584114

[112] Edholm ES Albertorio Saez LM Gill AL Gill SR Grayfer L Haynes N. Nonclassical MHC Class I-Dependent Invariant T Cells are Evolutionarily Conserved and Prominent From Early Development in Amphibians. Proc Natl Acad Sci USA (2013) 110:14342–7. doi: 10.4049/jimmunol.1500458 PMC3761575 23940320

[113] Edholm ES Grayfer L De Jesús Andino F Robert J. Nonclassical MHC-Restricted Invariant Vα6 T Cells are Critical for Efficient Early Innate Antiviral Immunity in the Amphibian *Xenopus Laevis* . J Immunol (2015) 195:576–86. doi: 10.4049/jimmunol.1500458 PMC4490974 26062996

[114] Edholm ESI De Jesús Andino F Yim J Woo K Robert J. Critical Role of an MHC Class I-Like/Innate-Like T Cell Immune Surveillance System in Host Defense Against Ranavirus (Frog Virus 3) Infection. Virus (2019) 11:330. doi: 10.3390/v11040330 PMC6521289 30959883

[115] Edholm ES Banach M Robert J. Evolution of Innate-Like T Cells and Their Selection by MHC Class I-Like Molecules. Immunogenetics (2016) 68:525–36. doi: 10.1007/s00251-016-0929-7 PMC5695035 27368412

[116] Zhou Y Fan Y LaPatra SE Ma J Xu J Meng Y. Protective Immunity of a *Pichia Pastoris* Expressed Recombinant Iridovirus Major Capsid Protein in the Chinese Giant Salamander, Andrias Davidianus. Vaccine (2015) 33:5662–9. doi: 10.1016/j.vaccine.2015.08.054 26303874

[117] Chen Z Li T Gao X Wang C Zhang Q. Protective Immunity Induced by DNA Vaccination Against Ranavirus Infection in Chinese Giant Salamander *Andrias Davidianus* . Viruses (2018) 10:52. doi: 10.3390/v10020052 PMC5850359 29364850

[118] Chinchar VG Yu KH Jancovich JK. The Molecular Biology of Frog Virus 3 and Other Iridoviruses Infecting Cold-Blooded Vertebrates. Viruses (2011) 3:1959–85. doi: 10.3390/v3101959 PMC3205390 22069524

[119] Jancovich JK Jacobs BL. Innate Immune Evasion Mediated by the Ambystoma Tigrinum Virus Eukaryotic Translation Initiation Factor 2alpha Homologue. J Virol (2011) 85:5061–9. doi: 10.1128/JVI.01488-10 PMC3126196 21389122

[120] Chen G Ward BM Yu KH Chinchar VG Robert L. Improved Knockout Methodology Reveals That Frog Virus 3 Mutants Lacking Either the 18K Immediate-Early Gene or the Truncated vIF-2 Gene are Defective for Replication and Growth *In Vivo* . J Virol (2011) 85:11131–8. doi: 10.1128/JVI.05589-11 PMC3194944 21865381

[121] De Jesús Andino F Grayfer L Chen G Chinchar VG Edholm E Robert J. Characterization of Frog Virus 3 Knockout Mutants Lacking Putative Virulence Genes. Virology (2015) 485:162–70. doi: 10.1016/j.virol.2015.07.011 PMC4619136 26264970

[122] Yu NT Ke F Zhang QY. *Andrias Davidianus* Ranavirus *1R* En**co**ding a Delayed-Early Protein Promotes Cell Proliferation by Driving Cell Cycle Progression Into S Phase. Acta Virol (2020) 64:10–9. doi: 10.4149/av_2020_102 32180414

[123] Yu NT Zhang QY. A Transmembrane Domain of *Andrias Davidianus* Ranavirus 13R is Crucial for Co-Localization to Endoplasmic Reticulum and Viromatrix. 3 Biotech (2019) 9:433. doi: 10.1007/s13205-019-1961-8 PMC6825229 31696038

